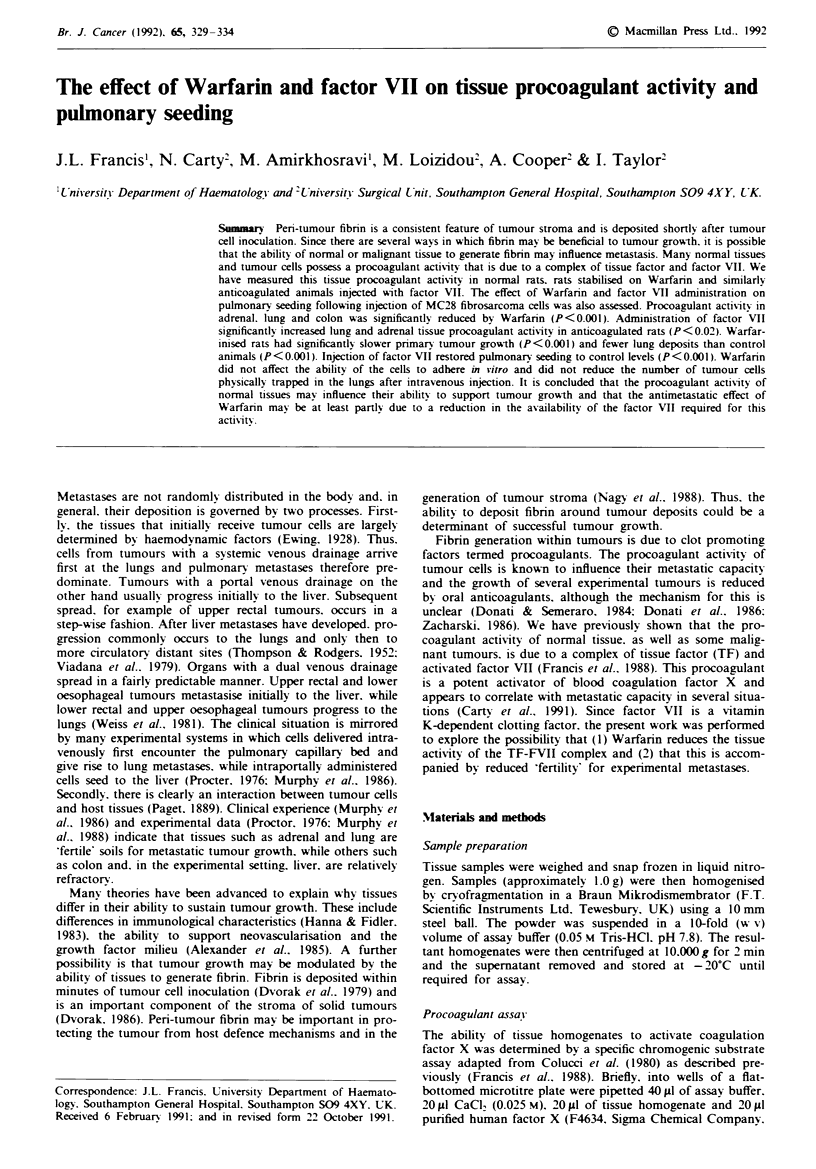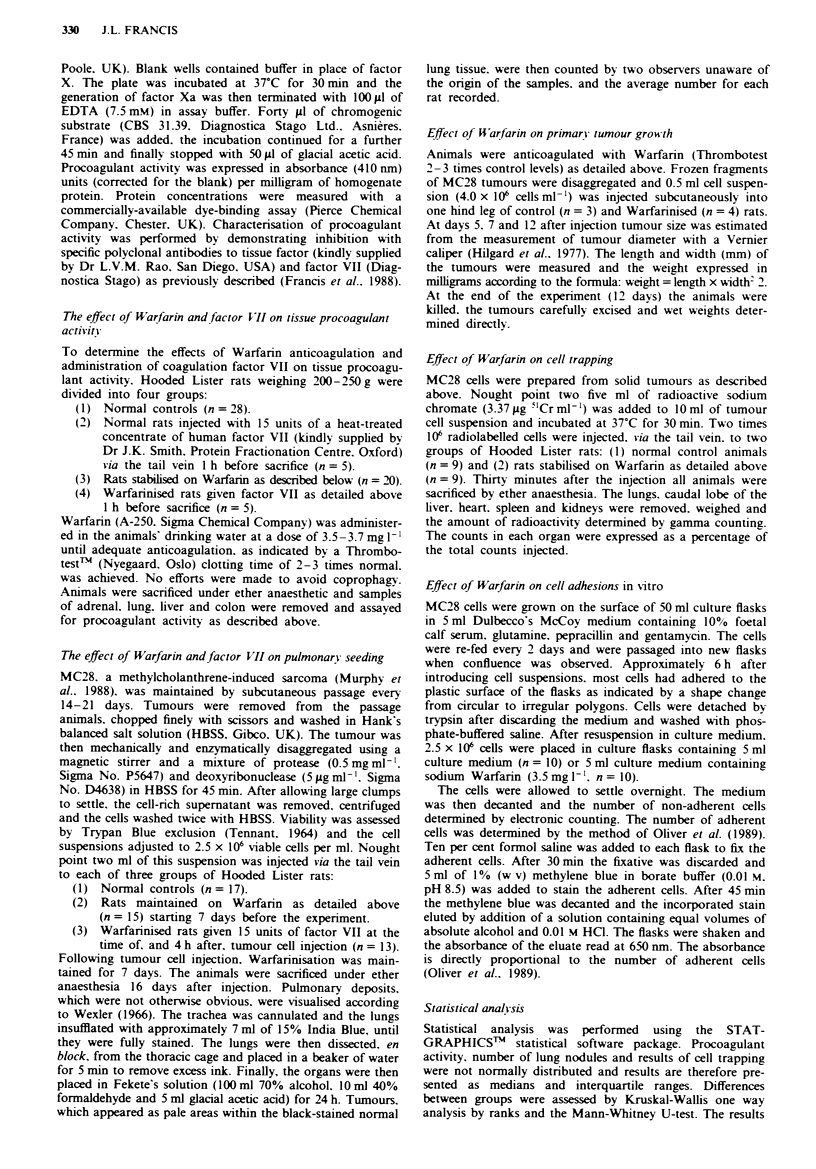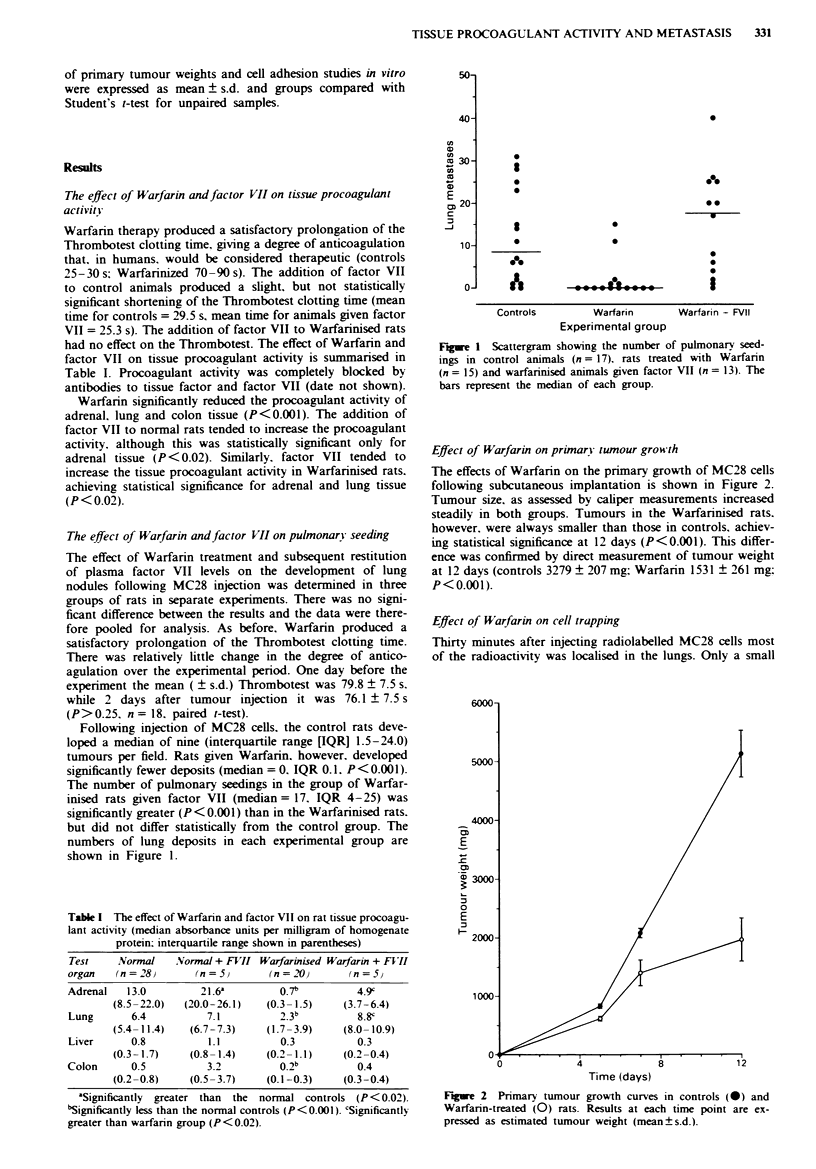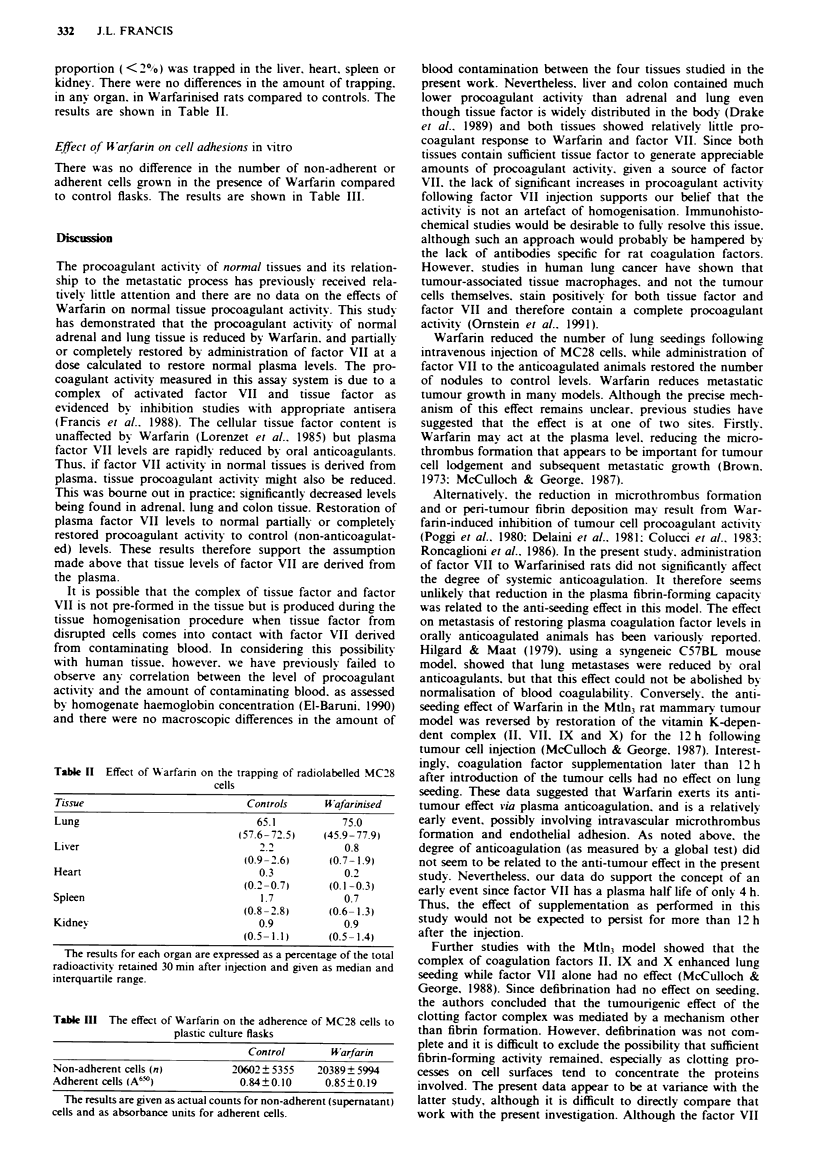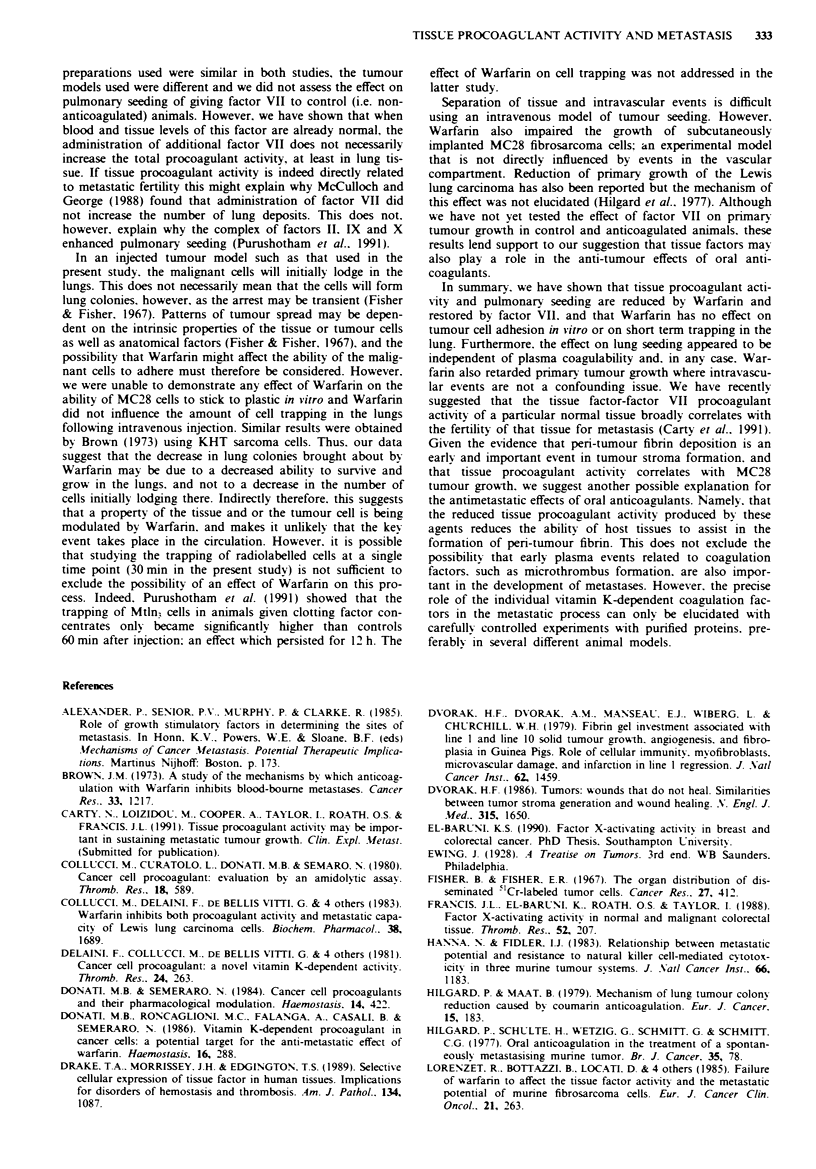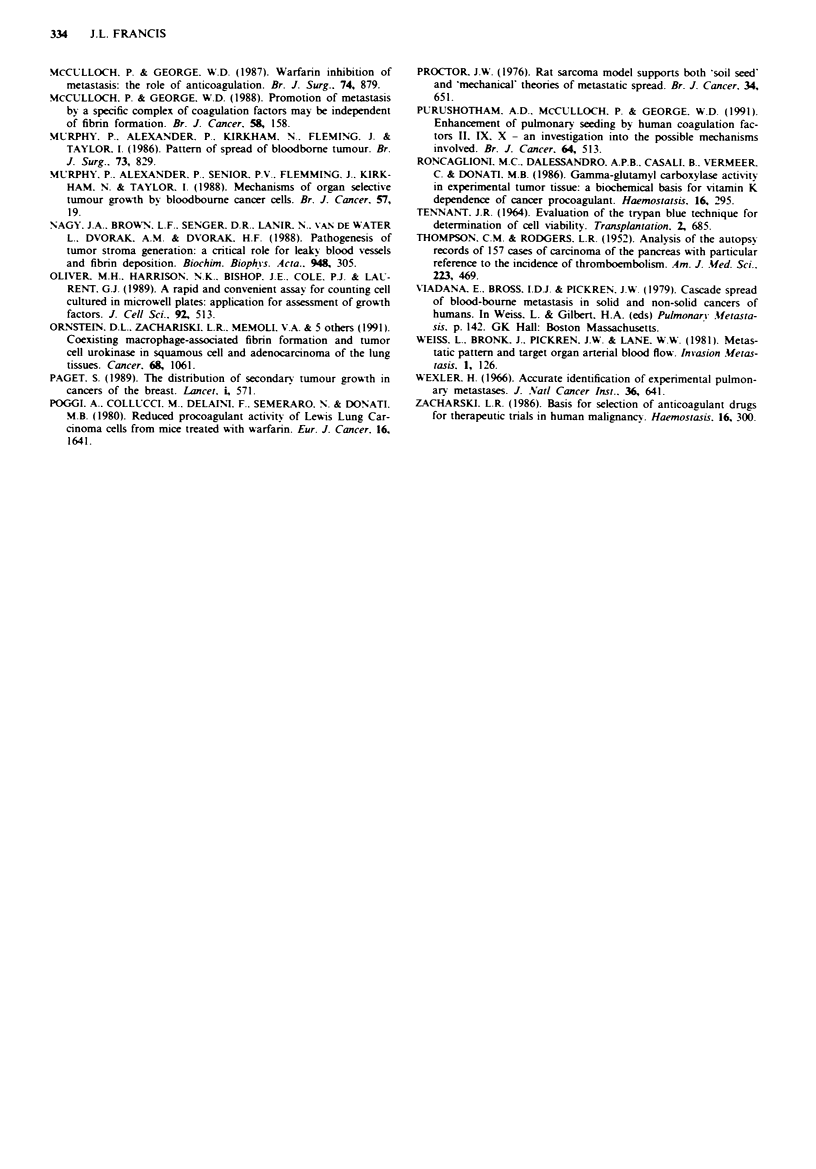# The effect of Warfarin and factor VII on tissue procoagulant activity and pulmonary seeding.

**DOI:** 10.1038/bjc.1992.67

**Published:** 1992-03

**Authors:** J. L. Francis, N. Carty, M. Amirkhosravi, M. Loizidou, A. Cooper, I. Taylor

**Affiliations:** University Department of Haematology, Southampton General Hospital, UK.

## Abstract

Peri-tumour fibrin is a consistent feature of tumour stroma and is deposited shortly after tumour cell inoculation. Since there are several ways in which fibrin may be beneficial to tumour growth, it is possible that the ability of normal or malignant tissue to generate fibrin may influence metastasis. Many normal tissues and tumour cells possess a procoagulant activity that is due to a complex of tissue factor and factor VII. We have measured this tissue procoagulant activity in normal rats, rats stabilised on Warfarin and similarly anticoagulated animals injected with factor VII. The effect of Warfarin and factor VII administration on pulmonary seeding following injection of MC28 fibrosarcoma cells was also assessed. Procoagulant activity in adrenal, lung and colon was significantly reduced by Warfarin (P less than 0.001). Administration of factor VII significantly increased lung and adrenal tissue procoagulant activity in anticoagulated rats (P less than 0.02). Warfarinised rats had significantly slower primary tumour growth (P less than 0.001) and fewer lung deposits than control animals (P less than 0.001). Injection of factor VII restored pulmonary seeding to control levels (P less than 0.001). Warfarin did not affect the ability of the cells to adhere in vitro and did not reduce the number of tumour cells physically trapped in the lungs after intravenous injection. It is concluded that the procoagulant activity of normal tissues may influence their ability to support tumour growth and that the antimetastatic effect of Warfarin may be at least partly due to a reduction in the availability of the factor VII required for this activity.


Br. J. Cancer (1992), 65, 329-334 © Macmillan Press Ltd., 1992

### The effect of Warfarin and factor VII on tissue procoagulant activity and pulmonary seeding

J.L. Francis[1], N. Carty[2], M. Amirkhosravi[1], M. Loizidou[2], A. Cooper[2] & I. Taylor[2]

[1]University Department of Haematology and [2]University Surgical Unit, Southampton General Hospital, Southampton SO9 4XY, UK.

**Summary** Peri-tumour fibrin is a consistent feature of tumour stroma and is deposited shortly after tumour cell inoculation. Since there are several ways in which fibrin may be beneficial to tumour growth, it is possible that the ability of normal or malignant tissue to generate fibrin may influence metastasis. Many normal tissues and tumour cells possess a procoagulant activity that is due to a complex of tissue factor and factor VII. We have measured this tissue procoagulant activity in normal rats, rats stabilised on Warfarin and similarly anticoagulated animals injected with factor VII. The effect of Warfarin and factor VII administration on pulmonary seeding following injection of MC28 fibrosarcoma cells was also assessed. Procoagulant activity in adrenal, lung and colon was significantly reduced by Warfarin ($P<0.001$). Administration of factor VII significantly increased lung and adrenal tissue procoagulant activity in anticoagulated rats ($P<0.02$). Warfarinised rats had significantly slower primary tumour growth ($P<0.001$) and fewer lung deposits than control animals ($P<0.001$). Injection of factor VII restored pulmonary seeding to control levels ($P<0.001$). Warfarin did not affect the ability of the cells to adhere *in vitro* and did not reduce the number of tumour cells physically trapped in the lungs after intravenous injection. It is concluded that the procoagulant activity of normal tissues may influence their ability to support tumour growth and that the antimetastatic effect of Warfarin may be at least partly due to a reduction in the availability of the factor VII required for this activity.

Metastases are not randomly distributed in the body and, in general, their deposition is governed by two processes. Firstly, the tissues that initially receive tumour cells are largely determined by haemodynamic factors (Ewing, 1928). Thus, cells from tumours with a systemic venous drainage arrive first at the lungs and pulmonary metastases therefore predominate. Tumours with a portal venous drainage on the other hand usually progress initially to the liver. Subsequent spread, for example of upper rectal tumours, occurs in a step-wise fashion. After liver metastases have developed, progression commonly occurs to the lungs and only then to more circulatory distant sites (Thompson & Rodgers, 1952; Viadana *et al.*, 1979). Organs with a dual venous drainage spread in a fairly predictable manner. Upper rectal and lower oesophageal tumours metastasise initially to the liver, while lower rectal and upper oesophageal tumours progress to the lungs (Weiss *et al.*, 1981). The clinical situation is mirrored by many experimental systems in which cells delivered intravenously first encounter the pulmonary capillary bed and give rise to lung metastases, while intraportally administered cells seed to the liver (Procter, 1976; Murphy *et al.*, 1986). Secondly, there is clearly an interaction between tumour cells and host tissues (Paget, 1889). Clinical experience (Murphy *et al.*, 1986) and experimental data (Proctor, 1976; Murphy *et al.*, 1988) indicate that tissues such as adrenal and lung are 'fertile' soils for metastatic tumour growth, while others such as colon and, in the experimental setting, liver, are relatively refractory.

Many theories have been advanced to explain why tissues differ in their ability to sustain tumour growth. These include differences in immunological characteristics (Hanna & Fidler, 1983), the ability to support neovascularisation and the growth factor milieu (Alexander *et al.*, 1985). A further possibility is that tumour growth may be modulated by the ability of tissues to generate fibrin. Fibrin is deposited within minutes of tumour cell inoculation (Dvorak *et al.*, 1979) and is an important component of the stroma of solid tumours (Dvorak, 1986). Peri-tumour fibrin may be important in protecting the tumour from host defence mechanisms and in the generation of tumour stroma (Nagy *et al.*, 1988). Thus, the ability to deposit fibrin around tumour deposits could be a determinant of successful tumour growth.

Fibrin generation within tumours is due to clot promoting factors termed procoagulants. The procoagulant activity of tumour cells is known to influence their metastatic capacity and the growth of several experimental tumours is reduced by oral anticoagulants, although the mechanism for this is unclear (Donati & Semeraro, 1984; Donati *et al.*, 1986; Zacharski, 1986). We have previously shown that the procoagulant activity of normal tissue, as well as some malignant tumours, is due to a complex of tissue factor (TF) and activated factor VII (Francis *et al.*, 1988). This procoagulant is a potent activator of blood coagulation factor X and appears to correlate with metastatic capacity in several situations (Carty *et al.*, 1991). Since factor VII is a vitamin K-dependent clotting factor, the present work was performed to explore the possibility that (1) Warfarin reduces the tissue activity of the TF-FVII complex and (2) that this is accompanied by reduced 'fertility' for experimental metastases.

### Materials and methods

### Sample preparation

Tissue samples were weighed and snap frozen in liquid nitrogen. Samples (approximately 1.0 g) were then homogenised by cryofragmentation in a Braun Mikrodismembrator (F.T. Scientific Instruments Ltd, Tewesbury, UK) using a 10 mm steel ball. The powder was suspended in a 10-fold (w v) volume of assay buffer (0.05 M Tris-HCl, pH 7.8). The resultant homogenates were then centrifuged at 10,000 *g* for 2 min and the supernatant removed and stored at −20°C until required for assay.

### Procoagulant assay

The ability of tissue homogenates to activate coagulation factor X was determined by a specific chromogenic substrate assay adapted from Colucci *et al.* (1980) as described previously (Francis *et al.*, 1988). Briefly, into wells of a flat-bottomed microtitre plate were pipetted 40 µl of assay buffer, 20 µl $CaCl_2$ (0.025 M), 20 µl of tissue homogenate and 20 µl purified human factor X (F4634, Sigma Chemical Company,

Correspondence: J.L. Francis, University Department of Haematology, Southampton General Hospital, Southampton SO9 4XY, UK.
Received 6 February 1991; and in revised form 22 October 1991.

Poole, UK). Blank wells contained buffer in place of factor X. The plate was incubated at 37°C for 30 min and the generation of factor Xa was then terminated with 100 µl of EDTA (7.5 mM) in assay buffer. Forty µl of chromogenic substrate (CBS 31.39, Diagnostica Stago Ltd., Asnières, France) was added, the incubation continued for a further 45 min and finally stopped with 50 µl of glacial acetic acid. Procoagulant activity was expressed in absorbance (410 nm) units (corrected for the blank) per milligram of homogenate protein. Protein concentrations were measured with a commercially-available dye-binding assay (Pierce Chemical Company, Chester, UK). Characterisation of procoagulant activity was performed by demonstrating inhibition with specific polyclonal antibodies to tissue factor (kindly supplied by Dr L.V.M. Rao, San Diego, USA) and factor VII (Diagnostica Stago) as previously described (Francis *et al.*, 1988).

*The effect of Warfarin and factor VII on tissue procoagulant activity*

To determine the effects of Warfarin anticoagulation and administration of coagulation factor VII on tissue procoagulant activity, Hooded Lister rats weighing 200–250 g were divided into four groups:

(1) Normal controls ($n = 28$).
(2) Normal rats injected with 15 units of a heat-treated concentrate of human factor VII (kindly supplied by Dr J.K. Smith, Protein Fractionation Centre, Oxford) *via* the tail vein 1 h before sacrifice ($n = 5$).
(3) Rats stabilised on Warfarin as described below ($n = 20$).
(4) Warfarinised rats given factor VII as detailed above 1 h before sacrifice ($n = 5$).

Warfarin (A-250, Sigma Chemical Company) was administered in the animals' drinking water at a dose of 3.5–3.7 mg $l^{-1}$ until adequate anticoagulation, as indicated by a Thrombotest™ (Nyegaard, Oslo) clotting time of 2–3 times normal, was achieved. No efforts were made to avoid coprophagy. Animals were sacrificed under ether anaesthetic and samples of adrenal, lung, liver and colon were removed and assayed for procoagulant activity as described above.

*The effect of Warfarin and factor VII on pulmonary seeding*

MC28, a methylcholanthrene-induced sarcoma (Murphy *et al.*, 1988), was maintained by subcutaneous passage every 14–21 days. Tumours were removed from the passage animals, chopped finely with scissors and washed in Hank's balanced salt solution (HBSS, Gibco, UK). The tumour was then mechanically and enzymatically disaggregated using a magnetic stirrer and a mixture of protease (0.5 mg $ml^{-1}$, Sigma No. P5647) and deoxyribonuclease (5 µg $ml^{-1}$, Sigma No. D4638) in HBSS for 45 min. After allowing large clumps to settle, the cell-rich supernatant was removed, centrifuged and the cells washed twice with HBSS. Viability was assessed by Trypan Blue exclusion (Tennant, 1964) and the cell suspensions adjusted to $2.5 \times 10^6$ viable cells per ml. Nought point two ml of this suspension was injected *via* the tail vein to each of three groups of Hooded Lister rats:

(1) Normal controls ($n = 17$).
(2) Rats maintained on Warfarin as detailed above ($n = 15$) starting 7 days before the experiment.
(3) Warfarinised rats given 15 units of factor VII at the time of, and 4 h after, tumour cell injection ($n = 13$).

Following tumour cell injection, Warfarinisation was maintained for 7 days. The animals were sacrificed under ether anaesthesia 16 days after injection. Pulmonary deposits, which were not otherwise obvious, were visualised according to Wexler (1966). The trachea was cannulated and the lungs insufflated with approximately 7 ml of 15% India Blue, until they were fully stained. The lungs were then dissected, *en block*, from the thoracic cage and placed in a beaker of water for 5 min to remove excess ink. Finally, the organs were then placed in Fekete's solution (100 ml 70% alcohol, 10 ml 40% formaldehyde and 5 ml glacial acetic acid) for 24 h. Tumours, which appeared as pale areas within the black-stained normal lung tissue, were then counted by two observers unaware of the origin of the samples, and the average number for each rat recorded.

*Effect of Warfarin on primary tumour growth*

Animals were anticoagulated with Warfarin (Thrombotest 2–3 times control levels) as detailed above. Frozen fragments of MC28 tumours were disaggregated and 0.5 ml cell suspension ($4.0 \times 10^6$ cells $ml^{-1}$) was injected subcutaneously into one hind leg of control ($n = 3$) and Warfarinised ($n = 4$) rats. At days 5, 7 and 12 after injection tumour size was estimated from the measurement of tumour diameter with a Vernier caliper (Hilgard *et al.*, 1977). The length and width (mm) of the tumours were measured and the weight expressed in milligrams according to the formula: weight = length × width$^2$/2. At the end of the experiment (12 days) the animals were killed, the tumours carefully excised and wet weights determined directly.

*Effect of Warfarin on cell trapping*

MC28 cells were prepared from solid tumours as described above. Nought point two five ml of radioactive sodium chromate (3.37 µg $^{51}$Cr $ml^{-1}$) was added to 10 ml of tumour cell suspension and incubated at 37°C for 30 min. Two times $10^6$ radiolabelled cells were injected, *via* the tail vein, to two groups of Hooded Lister rats: (1) normal control animals ($n = 9$) and (2) rats stabilised on Warfarin as detailed above ($n = 9$). Thirty minutes after the injection all animals were sacrificed by ether anaesthesia. The lungs, caudal lobe of the liver, heart, spleen and kidneys were removed, weighed and the amount of radioactivity determined by gamma counting. The counts in each organ were expressed as a percentage of the total counts injected.

*Effect of Warfarin on cell adhesions* in vitro

MC28 cells were grown on the surface of 50 ml culture flasks in 5 ml Dulbecco's McCoy medium containing 10% foetal calf serum, glutamine, pepracillin and gentamycin. The cells were re-fed every 2 days and were passaged into new flasks when confluence was observed. Approximately 6 h after introducing cell suspensions, most cells had adhered to the plastic surface of the flasks as indicated by a shape change from circular to irregular polygons. Cells were detached by trypsin after discarding the medium and washed with phosphate-buffered saline. After resuspension in culture medium, $2.5 \times 10^6$ cells were placed in culture flasks containing 5 ml culture medium ($n = 10$) or 5 ml culture medium containing sodium Warfarin (3.5 mg $l^{-1}$, $n = 10$).

The cells were allowed to settle overnight. The medium was then decanted and the number of non-adherent cells determined by electronic counting. The number of adherent cells was determined by the method of Oliver *et al.* (1989). Ten per cent formol saline was added to each flask to fix the adherent cells. After 30 min the fixative was discarded and 5 ml of 1% (w/v) methylene blue in borate buffer (0.01 M, pH 8.5) was added to stain the adherent cells. After 45 min the methylene blue was decanted and the incorporated stain eluted by addition of a solution containing equal volumes of absolute alcohol and 0.01 M HCl. The flasks were shaken and the absorbance of the eluate read at 650 nm. The absorbance is directly proportional to the number of adherent cells (Oliver *et al.*, 1989).

*Statistical analysis*

Statistical analysis was performed using the STATGRAPHICS™ statistical software package. Procoagulant activity, number of lung nodules and results of cell trapping were not normally distributed and results are therefore presented as medians and interquartile ranges. Differences between groups were assessed by Kruskal-Wallis one way analysis by ranks and the Mann-Whitney U-test. The results of primary tumour weights and cell adhesion studies *in vitro* were expressed as mean ± s.d. and groups compared with Student's *t*-test for unpaired samples.

### Results

### *The effect of Warfarin and factor VII on tissue procoagulant activity*

Warfarin therapy produced a satisfactory prolongation of the Thrombotest clotting time, giving a degree of anticoagulation that, in humans, would be considered therapeutic (controls 25–30 s; Warfarinized 70–90 s). The addition of factor VII to control animals produced a slight, but not statistically significant shortening of the Thrombotest clotting time (mean time for controls = 29.5 s, mean time for animals given factor VII = 25.3 s). The addition of factor VII to Warfarinised rats had no effect on the Thrombotest. The effect of Warfarin and factor VII on tissue procoagulant activity is summarised in Table I. Procoagulant activity was completely blocked by antibodies to tissue factor and factor VII (date not shown).

Warfarin significantly reduced the procoagulant activity of adrenal, lung and colon tissue ($P<0.001$). The addition of factor VII to normal rats tended to increase the procoagulant activity, although this was statistically significant only for adrenal tissue ($P<0.02$). Similarly, factor VII tended to increase the tissue procoagulant activity in Warfarinised rats, achieving statistical significance for adrenal and lung tissue ($P<0.02$).

### *The effect of Warfarin and factor VII on pulmonary seeding*

The effect of Warfarin treatment and subsequent restitution of plasma factor VII levels on the development of lung nodules following MC28 injection was determined in three groups of rats in separate experiments. There was no significant difference between the results and the data were therefore pooled for analysis. As before, Warfarin produced a satisfactory prolongation of the Thrombotest clotting time. There was relatively little change in the degree of anticoagulation over the experimental period. One day before the experiment the mean ( ± s.d.) Thrombotest was 79.8 ± 7.5 s, while 2 days after tumour injection it was 76.1 ± 7.5 s ($P>0.25$, $n = 18$, paired *t*-test).

Following injection of MC28 cells, the control rats developed a median of nine (interquartile range [IQR] 1.5–24.0) tumours per field. Rats given Warfarin, however, developed significantly fewer deposits (median = 0, IQR 0.1, $P<0.001$). The number of pulmonary seedings in the group of Warfarinised rats given factor VII (median = 17, IQR 4–25) was significantly greater ($P<0.001$) than in the Warfarinised rats, but did not differ statistically from the control group. The numbers of lung deposits in each experimental group are shown in Figure 1.

**Table I** The effect of Warfarin and factor VII on rat tissue procoagulant activity (median absorbance units per milligram of homogenate protein; interquartile range shown in parentheses)

| *Test organ* | *Normal (n = 28)* | *Normal + FVII (n = 5)* | *Warfarinised (n = 20)* | *Warfarin + FVII (n = 5)* |
|---|---|---|---|---|
| Adrenal | 13.0 | 21.6[a] | 0.7[b] | 4.9[c] |
| | (8.5–22.0) | (20.0–26.1) | (0.3–1.5) | (3.7–6.4) |
| Lung | 6.4 | 7.1 | 2.3[b] | 8.8[c] |
| | (5.4–11.4) | (6.7–7.3) | (1.7–3.9) | (8.0–10.9) |
| Liver | 0.8 | 1.1 | 0.3 | 0.3 |
| | (0.3–1.7) | (0.8–1.4) | (0.2–1.1) | (0.2–0.4) |
| Colon | 0.5 | 3.2 | 0.2[b] | 0.4 |
| | (0.2–0.8) | (0.5–3.7) | (0.1–0.3) | (0.3–0.4) |

[a]Significantly greater than the normal controls ($P<0.02$). [b]Significantly less than the normal controls ($P<0.001$). [c]Significantly greater than warfarin group ($P<0.02$).

**Figure 1** Scattergram showing the number of pulmonary seedings in control animals ($n = 17$), rats treated with Warfarin ($n = 15$) and warfarinised animals given factor VII ($n = 13$). The bars represent the median of each group.

### *Effect of Warfarin on primary tumour growth*

The effects of Warfarin on the primary growth of MC28 cells following subcutaneous implantation is shown in Figure 2. Tumour size, as assessed by caliper measurements increased steadily in both groups. Tumours in the Warfarinised rats, however, were always smaller than those in controls, achieving statistical significance at 12 days ($P<0.001$). This difference was confirmed by direct measurement of tumour weight at 12 days (controls 3279 ± 207 mg; Warfarin 1531 ± 261 mg; $P<0.001$).

### *Effect of Warfarin on cell trapping*

Thirty minutes after injecting radiolabelled MC28 cells most of the radioactivity was localised in the lungs. Only a small

**Figure 2** Primary tumour growth curves in controls (●) and Warfarin-treated (○) rats. Results at each time point are expressed as estimated tumour weight (mean ± s.d.).

proportion (<2%) was trapped in the liver, heart, spleen or kidney. There were no differences in the amount of trapping, in any organ, in Warfarinised rats compared to controls. The results are shown in Table II.

*Effect of Warfarin on cell adhesions* in vitro

There was no difference in the number of non-adherent or adherent cells grown in the presence of Warfarin compared to control flasks. The results are shown in Table III.

### Discussion

The procoagulant activity of *normal* tissues and its relationship to the metastatic process has previously received relatively little attention and there are no data on the effects of Warfarin on normal tissue procoagulant activity. This study has demonstrated that the procoagulant activity of normal adrenal and lung tissue is reduced by Warfarin, and partially or completely restored by administration of factor VII at a dose calculated to restore normal plasma levels. The procoagulant activity measured in this assay system is due to a complex of activated factor VII and tissue factor as evidenced by inhibition studies with appropriate antisera (Francis *et al.*, 1988). The cellular tissue factor content is unaffected by Warfarin (Lorenzet *et al.*, 1985) but plasma factor VII levels are rapidly reduced by oral anticoagulants. Thus, if factor VII activity in normal tissues is derived from plasma, tissue procoagulant activity might also be reduced. This was bourne out in practice; significantly decreased levels being found in adrenal, lung and colon tissue. Restoration of plasma factor VII levels to normal partially or completely restored procoagulant activity to control (non-anticoagulated) levels. These results therefore support the assumption made above that tissue levels of factor VII are derived from the plasma.

It is possible that the complex of tissue factor and factor VII is not pre-formed in the tissue but is produced during the tissue homogenisation procedure when tissue factor from disrupted cells comes into contact with factor VII derived from contaminating blood. In considering this possibility with human tissue, however, we have previously failed to observe any correlation between the level of procoagulant activity and the amount of contaminating blood, as assessed by homogenate haemoglobin concentration (El-Baruni, 1990) and there were no macroscopic differences in the amount of blood contamination between the four tissues studied in the present work. Nevertheless, liver and colon contained much lower procoagulant activity than adrenal and lung even though tissue factor is widely distributed in the body (Drake *et al.*, 1989) and both tissues showed relatively little procoagulant response to Warfarin and factor VII. Since both tissues contain sufficient tissue factor to generate appreciable amounts of procoagulant activity, given a source of factor VII, the lack of significant increases in procoagulant activity following factor VII injection supports our belief that the activity is not an artefact of homogenisation. Immunohistochemical studies would be desirable to fully resolve this issue, although such an approach would probably be hampered by the lack of antibodies specific for rat coagulation factors. However, studies in human lung cancer have shown that tumour-associated tissue macrophages, and not the tumour cells themselves, stain positively for both tissue factor and factor VII and therefore contain a complete procoagulant activity (Ornstein *et al.*, 1991).

Warfarin reduced the number of lung seedings following intravenous injection of MC28 cells, while administration of factor VII to the anticoagulated animals restored the number of nodules to control levels. Warfarin reduces metastatic tumour growth in many models. Although the precise mechanism of this effect remains unclear, previous studies have suggested that the effect is at one of two sites. Firstly, Warfarin may act at the plasma level, reducing the microthrombus formation that appears to be important for tumour cell lodgement and subsequent metastatic growth (Brown, 1973; McCulloch & George, 1987).

Alternatively, the reduction in microthrombus formation and or peri-tumour fibrin deposition may result from Warfarin-induced inhibition of tumour cell procoagulant activity (Poggi *et al.*, 1980; Delaini *et al.*, 1981; Colucci *et al.*, 1983; Roncaglioni *et al.*, 1986). In the present study, administration of factor VII to Warfarinised rats did not significantly affect the degree of systemic anticoagulation. It therefore seems unlikely that reduction in the plasma fibrin-forming capacity was related to the anti-seeding effect in this model. The effect on metastasis of restoring plasma coagulation factor levels in orally anticoagulated animals has been variously reported. Hilgard & Maat (1979), using a syngeneic C57BL mouse model, showed that lung metastases were reduced by oral anticoagulants, but that this effect could not be abolished by normalisation of blood coagulability. Conversely, the anti-seeding effect of Warfarin in the $Mtln_3$ rat mammary tumour model was reversed by restoration of the vitamin K-dependent complex (II, VII, IX and X) for the 12 h following tumour cell injection (McCulloch & George, 1987). Interestingly, coagulation factor supplementation later than 12 h after introduction of the tumour cells had no effect on lung seeding. These data suggested that Warfarin exerts its anti-tumour effect *via* plasma anticoagulation, and is a relatively early event, possibly involving intravascular microthrombus formation and endothelial adhesion. As noted above, the degree of anticoagulation (as measured by a global test) did not seem to be related to the anti-tumour effect in the present study. Nevertheless, our data do support the concept of an early event since factor VII has a plasma half life of only 4 h. Thus, the effect of supplementation as performed in this study would not be expected to persist for more than 12 h after the injection.

Further studies with the $Mtln_3$ model showed that the complex of coagulation factors II, IX and X enhanced lung seeding while factor VII alone had no effect (McCulloch & George, 1988). Since defibrination had no effect on seeding, the authors concluded that the tumourigenic effect of the clotting factor complex was mediated by a mechanism other than fibrin formation. However, defibrination was not complete and it is difficult to exclude the possibility that sufficient fibrin-forming activity remained, especially as clotting processes on cell surfaces tend to concentrate the proteins involved. The present data appear to be at variance with the latter study, although it is difficult to directly compare that work with the present investigation. Although the factor VII

**Table II** Effect of Warfarin on the trapping of radiolabelled MC28 cells

| *Tissue* | *Controls* | *Wafarinised* |
|---|---|---|
| Lung | 65.1 | 75.0 |
| | (57.6–72.5) | (45.9–77.9) |
| Liver | 2.2 | 0.8 |
| | (0.9–2.6) | (0.7–1.9) |
| Heart | 0.3 | 0.2 |
| | (0.2–0.7) | (0.1–0.3) |
| Spleen | 1.7 | 0.7 |
| | (0.8–2.8) | (0.6–1.3) |
| Kidney | 0.9 | 0.9 |
| | (0.5–1.1) | (0.5–1.4) |

The results for each organ are expressed as a percentage of the total radioactivity retained 30 min after injection and given as median and interquartile range.

**Table III** The effect of Warfarin on the adherence of MC28 cells to plastic culture flasks

| | *Control* | *Warfarin* |
|---|---|---|
| Non-adherent cells (*n*) | 20602±5355 | 20389±5994 |
| Adherent cells ($A^{650}$) | 0.84±0.10 | 0.85±0.19 |

The results are given as actual counts for non-adherent (supernatant) cells and as absorbance units for adherent cells.

preparations used were similar in both studies, the tumour models used were different and we did not assess the effect on pulmonary seeding of giving factor VII to control (i.e. non-anticoagulated) animals. However, we have shown that when blood and tissue levels of this factor are already normal, the administration of additional factor VII does not necessarily increase the total procoagulant activity, at least in lung tissue. If tissue procoagulant activity is indeed directly related to metastatic fertility this might explain why McCulloch and George (1988) found that administration of factor VII did not increase the number of lung deposits. This does not, however, explain why the complex of factors II, IX and X enhanced pulmonary seeding (Purushotham *et al.*, 1991).

In an injected tumour model such as that used in the present study, the malignant cells will initially lodge in the lungs. This does not necessarily mean that the cells will form lung colonies, however, as the arrest may be transient (Fisher & Fisher, 1967). Patterns of tumour spread may be dependent on the intrinsic properties of the tissue or tumour cells as well as anatomical factors (Fisher & Fisher, 1967), and the possibility that Warfarin might affect the ability of the malignant cells to adhere must therefore be considered. However, we were unable to demonstrate any effect of Warfarin on the ability of MC28 cells to stick to plastic *in vitro* and Warfarin did not influence the amount of cell trapping in the lungs following intravenous injection. Similar results were obtained by Brown (1973) using KHT sarcoma cells. Thus, our data suggest that the decrease in lung colonies brought about by Warfarin may be due to a decreased ability to survive and grow in the lungs, and not to a decrease in the number of cells initially lodging there. Indirectly therefore, this suggests that a property of the tissue and or the tumour cell is being modulated by Warfarin, and makes it unlikely that the key event takes place in the circulation. However, it is possible that studying the trapping of radiolabelled cells at a single time point (30 min in the present study) is not sufficient to exclude the possibility of an effect of Warfarin on this process. Indeed, Purushotham *et al.* (1991) showed that the trapping of $Mtln_3$ cells in animals given clotting factor concentrates only became significantly higher than controls 60 min after injection; an effect which persisted for 12 h. The effect of Warfarin on cell trapping was not addressed in the latter study.

Separation of tissue and intravascular events is difficult using an intravenous model of tumour seeding. However, Warfarin also impaired the growth of subcutaneously implanted MC28 fibrosarcoma cells; an experimental model that is not directly influenced by events in the vascular compartment. Reduction of primary growth of the Lewis lung carcinoma has also been reported but the mechanism of this effect was not elucidated (Hilgard *et al.*, 1977). Although we have not yet tested the effect of factor VII on primary tumour growth in control and anticoagulated animals, these results lend support to our suggestion that tissue factors may also play a role in the anti-tumour effects of oral anticoagulants.

In summary, we have shown that tissue procoagulant activity and pulmonary seeding are reduced by Warfarin and restored by factor VII, and that Warfarin has no effect on tumour cell adhesion *in vitro* or on short term trapping in the lung. Furthermore, the effect on lung seeding appeared to be independent of plasma coagulability and, in any case, Warfarin also retarded primary tumour growth where intravascular events are not a confounding issue. We have recently suggested that the tissue factor-factor VII procoagulant activity of a particular normal tissue broadly correlates with the fertility of that tissue for metastasis (Carty *et al.*, 1991). Given the evidence that peri-tumour fibrin deposition is an early and important event in tumour stroma formation, and that tissue procoagulant activity correlates with MC28 tumour growth, we suggest another possible explanation for the antimetastatic effects of oral anticoagulants. Namely, that the reduced tissue procoagulant activity produced by these agents reduces the ability of host tissues to assist in the formation of peri-tumour fibrin. This does not exclude the possibility that early plasma events related to coagulation factors, such as microthrombus formation, are also important in the development of metastases. However, the precise role of the individual vitamin K-dependent coagulation factors in the metastatic process can only be elucidated with carefully controlled experiments with purified proteins, preferably in several different animal models.

### References

ALEXANDER, P., SENIOR, P.V., MURPHY, P. & CLARKE, R. (1985). Role of growth stimulatory factors in determining the sites of metastasis. In Honn, K.V., Powers, W.E. & Sloane, B.F. (eds) *Mechanisms of Cancer Metastasis. Potential Therapeutic Implications*. Martinus Nijhoff: Boston, p. 173.

BROWN, J.M. (1973). A study of the mechanisms by which anticoagulation with Warfarin inhibits blood-bourne metastases. *Cancer Res.*, **33**, 1217.

CARTY, N., LOIZIDOU, M., COOPER, A., TAYLOR, I., ROATH, O.S. & FRANCIS, J.L. (1991). Tissue procoagulant activity may be important in sustaining metastatic tumour growth. *Clin. Expl. Metast.* (Submitted for publication).

COLLUCCI, M., CURATOLO, L., DONATI, M.B. & SEMARO, N. (1980). Cancer cell procoagulant: evaluation by an amidolytic assay. *Thromb. Res.*, **18**, 589.

COLLUCCI, M., DELAINI, F., DE BELLIS VITTI, G. & 4 others (1983). Warfarin inhibits both procoagulant activity and metastatic capacity of Lewis lung carcinoma cells. *Biochem. Pharmacol.*, **38**, 1689.

DELAINI, F., COLLUCCI, M., DE BELLIS VITTI, G. & 4 others (1981). Cancer cell procoagulant: a novel vitamin K-dependent activity. *Thromb. Res.*, **24**, 263.

DONATI, M.B. & SEMERARO, N. (1984). Cancer cell procoagulants and their pharmacological modulation. *Haemostasis*, **14**, 422.

DONATI, M.B., RONCAGLIONI, M.C., FALANGA, A., CASALI, B. & SEMERARO, N. (1986). Vitamin K-dependent procoagulant in cancer cells: a potential target for the anti-metastatic effect of warfarin. *Haemostasis*, **16**, 288.

DRAKE, T.A., MORRISSEY, J.H. & EDGINGTON, T.S. (1989). Selective cellular expression of tissue factor in human tissues. Implications for disorders of hemostasis and thrombosis. *Am. J. Pathol.*, **134**, 1087.

DVORAK, H.F., DVORAK, A.M., MANSEAU, E.J., WIBERG, L. & CHURCHILL, W.H. (1979). Fibrin gel investment associated with line 1 and line 10 solid tumour growth, angiogenesis, and fibroplasia in Guinea Pigs. Role of cellular immunity, myofibroblasts, microvascular damage, and infarction in line 1 regression. *J. Natl Cancer Inst.*, **62**, 1459.

DVORAK, H.F. (1986). Tumors: wounds that do not heal. Similarities between tumor stroma generation and wound healing. *N. Engl. J. Med.*, **315**, 1650.

EL-BARUNI, K.S. (1990). Factor X-activating activity in breast and colorectal cancer. PhD Thesis, Southampton University.

EWING, J. (1928). *A Treatise on Tumors*. 3rd end. WB Saunders, Philadelphia.

FISHER, B. & FISHER, E.R. (1967). The organ distribution of disseminated $^{51}$Cr-labeled tumor cells. *Cancer Res.*, **27**, 412.

FRANCIS, J.L., EL-BARUNI, K., ROATH, O.S. & TAYLOR, I. (1988). Factor X-activating activity in normal and malignant colorectal tissue. *Thromb. Res.*, **52**, 207.

HANNA, N. & FIDLER, I.J. (1983). Relationship between metastatic potential and resistance to natural killer cell-mediated cytotoxicity in three murine tumour systems. *J. Natl Cancer Inst.*, **66**, 1183.

HILGARD, P. & MAAT, B. (1979). Mechanism of lung tumour colony reduction caused by coumarin anticoagulation. *Eur. J. Cancer*, **15**, 183.

HILGARD, P., SCHULTE, H., WETZIG, G., SCHMITT, G. & SCHMITT, C.G. (1977). Oral anticoagulation in the treatment of a spontaneously metastasising murine tumor. *Br. J. Cancer*, **35**, 78.

LORENZET, R., BOTTAZZI, B., LOCATI, D. & 4 others (1985). Failure of warfarin to affect the tissue factor activity and the metastatic potential of murine fibrosarcoma cells. *Eur. J. Cancer Clin. Oncol.*, **21**, 263.

McCULLOCH, P. & GEORGE, W.D. (1987). Warfarin inhibition of metastasis: the role of anticoagulation. *Br. J. Surg.*, **74**, 879.

McCULLOCH, P. & GEORGE, W.D. (1988). Promotion of metastasis by a specific complex of coagulation factors may be independent of fibrin formation. *Br. J. Cancer*, **58**, 158.

MURPHY, P., ALEXANDER, P., KIRKHAM, N., FLEMING, J. & TAYLOR, I. (1986). Pattern of spread of bloodborne tumour. *Br. J. Surg.*, **73**, 829.

MURPHY, P., ALEXANDER, P., SENIOR, P.V., FLEMMING, J., KIRKHAM, N. & TAYLOR, I. (1988). Mechanisms of organ selective tumour growth by bloodbourne cancer cells. *Br. J. Cancer*, **57**, 19.

NAGY, J.A., BROWN, L.F., SENGER, D.R., LANIR, N., VAN DE WATER L., DVORAK, A.M. & DVORAK, H.F. (1988). Pathogenesis of tumor stroma generation: a critical role for leaky blood vessels and fibrin deposition. *Biochim. Biophys. Acta.*, **948**, 305.

OLIVER, M.H., HARRISON, N.K., BISHOP, J.E., COLE, P.J. & LAURENT, G.J. (1989). A rapid and convenient assay for counting cell cultured in microwell plates: application for assessment of growth factors. *J. Cell Sci.*, **92**, 513.

ORNSTEIN, D.L., ZACHARISKI, L.R., MEMOLI, V.A. & 5 others (1991). Coexisting macrophage-associated fibrin formation and tumor cell urokinase in squamous cell and adenocarcinoma of the lung tissues. *Cancer*, **68**, 1061.

PAGET, S. (1989). The distribution of secondary tumour growth in cancers of the breast. *Lancet*, **i**, 571.

POGGI, A., COLLUCCI, M., DELAINI, F., SEMERARO, N. & DONATI, M.B. (1980). Reduced procoagulant activity of Lewis Lung Carcinoma cells from mice treated with warfarin. *Eur. J. Cancer*, **16**, 1641.

PROCTOR, J.W. (1976). Rat sarcoma model supports both 'soil seed' and 'mechanical' theories of metastatic spread. *Br. J. Cancer*, **34**, 651.

PURUSHOTHAM, A.D., McCULLOCH, P. & GEORGE, W.D. (1991). Enhancement of pulmonary seeding by human coagulation factors II, IX, X – an investigation into the possible mechanisms involved. *Br. J. Cancer*, **64**, 513.

RONCAGLIONI, M.C., DALESSANDRO, A.P.B., CASALI, B., VERMEER, C. & DONATI, M.B. (1986). Gamma-glutamyl carboxylase activity in experimental tumor tissue: a biochemical basis for vitamin K dependence of cancer procoagulant. *Haemostatsis*, **16**, 295.

TENNANT, J.R. (1964). Evaluation of the trypan blue technique for determination of cell viability. *Transplantation*, **2**, 685.

THOMPSON, C.M. & RODGERS, L.R. (1952). Analysis of the autopsy records of 157 cases of carcinoma of the pancreas with particular reference to the incidence of thromboembolism. *Am. J. Med. Sci.*, **223**, 469.

VIADANA, E., BROSS, I.D.J. & PICKREN, J.W. (1979). Cascade spread of blood-bourne metastasis in solid and non-solid cancers of humans. In Weiss, L. & Gilbert, H.A. (eds) *Pulmonary Metastasis*, p. 142. GK Hall: Boston Massachusetts.

WEISS, L., BRONK, J., PICKREN, J.W. & LANE, W.W. (1981). Metastatic pattern and target organ arterial blood flow. *Invasion Metastasis*, **1**, 126.

WEXLER, H. (1966). Accurate identification of experimental pulmonary metastases. *J. Natl Cancer Inst.*, **36**, 641.

ZACHARSKI, L.R. (1986). Basis for selection of anticoagulant drugs for therapeutic trials in human malignancy. *Haemostasis*, **16**, 300.